# The hydrothermal synthesis of blue-emitting boron-doped CQDs and its application for improving the photovoltaic parameters of organic solar cell

**DOI:** 10.3906/kim-2104-14

**Published:** 2021-08-02

**Authors:** Tuğbahan YILMAZ

**Affiliations:** Department of Physics, Faculty of Science, Selcuk University, Konya, Turkey

**Keywords:** Additive material, boron doped CQDs, organic solar cell (OSC), P3HT: PCBM blend

## Abstract

In this work, boron-doped CQDs (B-CQDs) were synthesized by using boric acid, urea, and citric acid via hydrothermal method to use as a novel additive material for poly(3-hexylthiophene-2,5-diyl) (P3HT):[6,6]-phenyl-C61-butyric acid methyl ester (PCBM) based organic solar cells. The OSCs with an inverted device structure were fabricated on titanium dioxide (TiO_2_) thin film. It was showed the crystallinity, morphological and optical properties of P3HT:PCBM films improved after B-CQDs additive. The best performance was obtained to be a 39.65% of FF, a 546 mV of V_oc_, an 8.606 mA cm^−2^ of J_sc_ after 3 vol.% B-CQDs addition in P3HT:PCBM blend. The power conversion efficiency (PCE) enhanced from 1.72% (non-doped device) to 2.33% (3% of B-CQDs) (a ~ 35% increase). The obtained results provided that B-CQDs are promising materials to improve the performance of solar cell applications. The novelty of this work is to improve the performance of OSCs using cost-effective and eco-friendly Boron CQDs as an additive. Besides, to achieve a good PCE of OSCs, it is necessary for utilized clean and green energy.

## 1. Introduction

Organic solar cells (OSCs) have introduced some advantages such as large-area, low-cost, flexibility, light weight, easy fabrication *via* solution process; therefore, OSCs are highly attracted in renewable energy application [1,2]. The most investigated OSCs device structure is based on bulk heterojunction (BHJ), a continuous interpenetrating network of semiconductor materials, and fullerene derivative [2]. As a photoactive layer, poly (3-hexylthiophene-2,5-diyl) (P3HT) and (6, 6)-phenyl C61 butyric acid methyl ester (PCBM) are widely utilized in literature [3,4]. Over the last decade, researchers have reached a PCE of 18.69% with bulk heterojunction OSCs [5,6]. Although the solar cell efficiency has improved, further studies are needed to improve fill factor (FF) [7], short-circuit current density (J_sc_) [8] and open-circuit voltage (V_oc_) [9]. There are many approaches to enhance OSCs efficiency, including synthesis of low bandgap donor materials [10], optimization of device structure [11], improving the morphology of photoactive layer [12], using different fabrication techniques [13], interface modification [14], and incorporating additives in the active layer [15]. Within these approaches, the incorporation of additives is one of the versatile techniques to improve the power conversion efficiency of OSCs through the enhancement of morphology of photoactive layer at the nanoscale. Up to now, small molecules, metallic nanoparticles, solvents, and quantum dots have been studied as additives [16–20]. However, there is a lack of studies related to the incorporation of additives of QDs into active layer. On the other hand, most studies have centred on the using QDs based on cadmium sulphide (CdS), cadmium selenide (CdSe), cadmium telluride (CdTe), and lead sulphide (PbS) [21]. In addition, carbon based QDs have been studied to enhance the power conversion efficiency of OSCs [22,23].

Carbon quantum dots (CQDs) have caught attention with regard to its advantages such as a high carrier mobility, high conductivity, a wide absorption range, a narrow emission range, and stability [24]. Being able to control the size of CQDs can make possible the adjustment of the energy levels of the valence band and conduction band of CQDs, which can lead to use in different applications [25–27]. Furthermore, a number of techniques for the synthesis of CQDs have been demonstrated, including hydrothermal, microwave, laser ablation, arc discharge, acidic oxidation, electrochemistry, pyrolysis techniques [23,28–31]. CQDs are a great candidate for optoelectronic applications, including lighting emitting diodes (LEDs), field effect transistor (FETs), solar cells (SCs), and sensors due to these unique properties [23,32–35].

Recently, heteroatom doped CQDs such as boron (B), nitrogen (N), phosphor (P), and fluorine (F) have been synthesized and applied as an additive for optoelectronic devices [22,36]. The addition of CQDs is an effective pathway to control and improve the photoluminescence (PL) properties used to measure quantum yield [37]. Notwithstanding, there are limited studies with regard to doped CQDs, the studies so far have showed that the efficiency of photoluminescence increased after boron doped CQDs (B-CQDs) [38,39]. Additionally, similarly N doped CQDs, boron can cause to form good crystalline structure of CQDs, which is attributed to improving conductivity [40]. On the other hand, B-CQDs are good candidates for organic solar cell applications due to its excellent stability against storage, UV illumination time, temperature, and pH value [41]. In light of the previous studies, using B-CQDs could be one of pathway to enhance the photovoltaic performance of OSCs.

Herein, boron doped CQDs synthesis was achieved by hydrothermal method to use as an additive into the P3HT:PCBM blend. B-CQDs were introduced with various volumes into P3HT:PCBM blend to improve energy harvesting properties, crystal structure, and charge transport properties of photoactive layer. Thanks to improved morphology, better crystallinity, effective charge transfer, phase separation, and interchain interaction of photoactive layer could be obtained, which essentially ushers to increase PCE. The results showed that the morphological and optical properties of P3HT:PCBM films and its crystallinity improved after the addition of B-CQDs. Compared to non-doped films, PCE increased from 1.72% (non-doped device) to 2.33% (%3 of B-CQDs additive). Additionally, a good crystalline structure of P3HT:PCBM layer was obtained after addition of B-CQDs of a good, which leads to increase PCE. After 3 vol.% of B-CQDs additive, the best photovoltaic parameter was achieved the PCE 2.33% with a 39.65% of FF, a 546 mV of V_oc_, an 8.606 mA cm^−2^ of J_sc_. The results clearly indicated that doping B-CQDs into P3HT:PCBM blend improved the solar cell performance.

## 2. Experimental

### 2.1. Synthesis of B-CQDs

**Figure 1:**
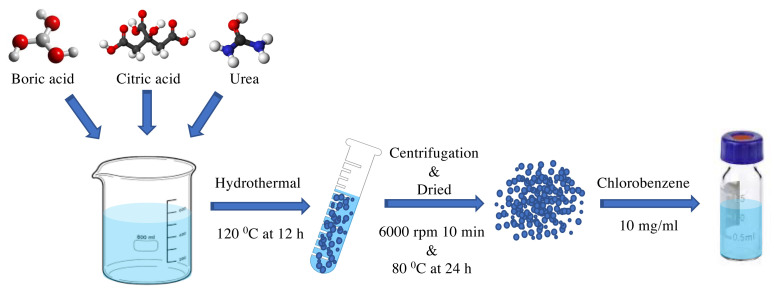
Synthesis process of B-CQDs.

B-CQDs synthesis procedure is shown in Figure 1. In a typical procedure, boric acid (4 g) (Merck), urea (4 g) (Sigma-Aldrich, 99%), and citric acid (4 g) (Sigma-Aldrich, 99%) were dissolved in deionized (DI) water (80 mL) [42]. Boric acid was used as boron source, while urea and citric acid were used as carbon source.

The prepared solution was mixed and transferred into stainless-steel autoclave for hydrothermal synthesis of B-CQDs at 120 °C for 12 h and then cooled to room temperature. Afterwards, the obtained solution was centrifugated at 6000 rpm for 10 min. After the excess water had been removed, remaining solid particles were dried in the oven at 80 °C for 24 h. B-CQDs solution was prepared by dispersing of certain amount of B-CQDs (10 mg/mL) in chlorobenzene.

### 2.2. Device fabrication

**Figure 2:**
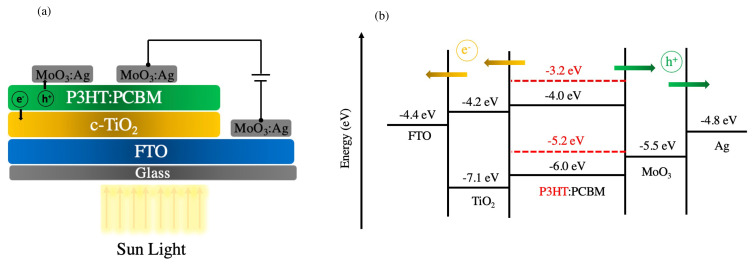
The energy conversion mechanism and energy levels of fabricated OSCs.

The structure of OSCs and corresponding energy level diagram are shown in Figure 2(a)&(b). Fluorine-doped tin oxide coated substrates (1.5 cm × 1.5 cm) were cleaned by diluted Hellmanex detergent solution (Sigma Aldrich, Hellmanex III), deionized water (DI), acetone, and isopropanol in an ultrasonic cleaner for 10 min, respectively. To remove organic traces, cleaned FTO surfaces were subjected to oxygen plasma treatment using a plasma cleaner (Diener Plasma System Femto PCCE-Plasma Cleaner) for 5 min after dried with N_2_. To form a compact-TiO_2_ (c-TiO_2_) layer, cleaned FTO surfaces were spin-coated by solution mixture of titanium (IV) isopropoxide (99.9%, Sigma-Aldrich) and acetyl acetone (99.5%, Sigma-Aldrich) with absolute ethanol as a precursor solution with a spinning speed of 3000 rpm for 20 s, following 2500 rpm for 20 se, and then all TiO_2_ coated substrates were left on hot plate at 110 °C for 10 min. Afterwards, sintering of FTO with TiO_2_ substrates was completed at 450 °C for 1 h in air. Then, all substrates were cooled down at room temperature before the active layer deposition.

To obtain an active layer, P3HT (Sigma-Aldrich):PCBM (Lumtec) blend solution was dissolved a 40 mg/mL in chloroform (CF): chlorobenzene (CB) (1:1) at 1.0:0.6 weight ratio, and then the prepared P3HT:PCBM solution was stirred at 70 °C overnight. The B-CQDs particles were dissolved in chlorobenzene at a 10 mg/mL to prepare dopant solution. Subsequently, prepared-P3HT: PCBM solution was doped with three various concentrations of B-CQDs solution (1 vol.%, 3 vol.% and 5 vol.%) after P3HT: PCBM solution filtered by polytetrafluoroethylene (PTFE) filter with 0.22 μm. The P3HT:PCBM solution with non-doped and doped was deposited on the c-TiO_2_ layer by spin-coating at 2200 rpm for 30 s, followed by 2500 rpm for 30 s. Then all coated substrates were heated at 160 °C for 10 min. To finalize the device fabrication, 10 nm thickness MoO_3_ and 80 nm thickness Ag electrodes were deposited by thermal under about 10^−6^ mbar vacuum pressure through a shadow mask (0.023 cm^2^ active area).

### 2.3. Characterization

The photoluminescent (PL) spectra of B-CQDs solution was taken by photoluminescent spectrometer (Perkin Elmer LS-55) with 350 nm excitation wavelength. The structure of B-CQDs was analysed by Transmission Electron Microscope (TEM, JEOL, Akishimashi, Tokyo, Japan) operated at 200 kV. The optical properties of B-CQDs solution and non-doped and doped B-CQDs P3HT:PCBM films were analysed by an ultraviolet-visible (UV–Vis) absorption spectrometer (Biochrom Libra S22) from 250 nm to 550 and 300 nm to 800 nm, respectively. The thickness of thin films was measured using a profilometer (NanoMap-LS). The morphology of non-doped and doped with B-CQDs P3HT:PCBM films was examined using an Atomic Force Microscope (AFM, NT-MDT NTEGRA Solaris). AFM images were taken in “tapping mode”. To understand the influence of the addition of B-CQDs on P3HT:PCBM film crystallization, all P3HT:PCBM layer with non-doped and doped B-CQDs were analysed by X-ray diffraction spectrometer (XRD, Bruker Advance D8) with Cu kα radiation at a wavelength of 1.5406 Å at 40 kV. The presence of boron element was measured The Scanning Electron Microscopy with Energy Dispersive X-Ray (SEM-EDX, Zeiss EVO LS 10) analysis. The current density-voltage (*J–V*) curves of the fabricated OPVs were determined by ATLAS solar simulator using a Keithley 2400 Source under illumination of a simulated sunlight (AM 1.5, 80 mW cm^−2^) in the glovebox.

## 3. Results and discussion

### 3.1. Structural, optical and morphological characterization of B-CQDs

**Figure 3:**
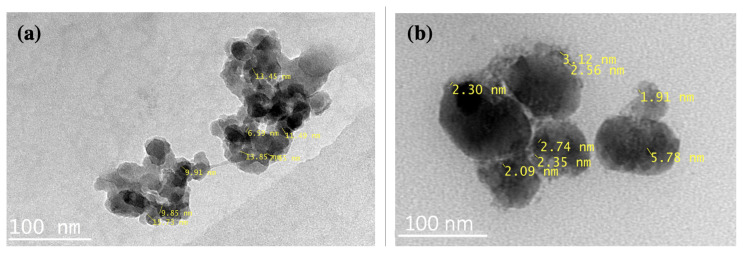
The TEM images of B-CQDs.

It is clear from Figure 3(a), B-CQDs shows a uniform distribution. TEM image indicated that B-CQDs are mostly of spherical form and well-dispersed as shown in Figure 3(b). In addition, B-CQDs were measured with a mean diameter of nearly 10 nm. Particle sizes are seen to be between less than 6 nm after zoomed into TEM image, which matches with our previous study [43].

**Figure 4:**
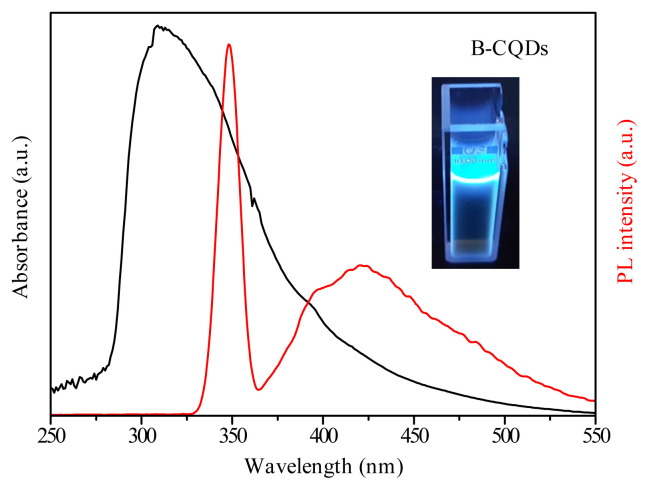
The measured UV-Vis absorption spectra (black) and photoluminescence (PL) spectra (red) of B-CQDs under the excitation wavelength of 350 nm [Inset: Photographs of B-CQDs under UV-light].

For further details in the optical and structural properties of B-CQDs, UV-Vis absorption spectra (black line) and PL spectra (red line) were taken as shown Figure 4. The UV-Vis absorption spectra (black line) indicated that the peaks observed at around 330 nm. The λ_max_ is attributed to n–π* transition of carbonyl bonds [30,44]. In addition, according to PL spectrum (red line), a slightly strong emission was observed at around 425 nm while excitation at 350 nm as well as correspond to bright blue light under UV light was shown in the inset of Figure 4. Further, the results clearly showed that B-CQDs has a high fluorescent property in the blue spectral region, which is associated with the optical properties of the quantum dots [31,45, 46].

**Figure 5:**
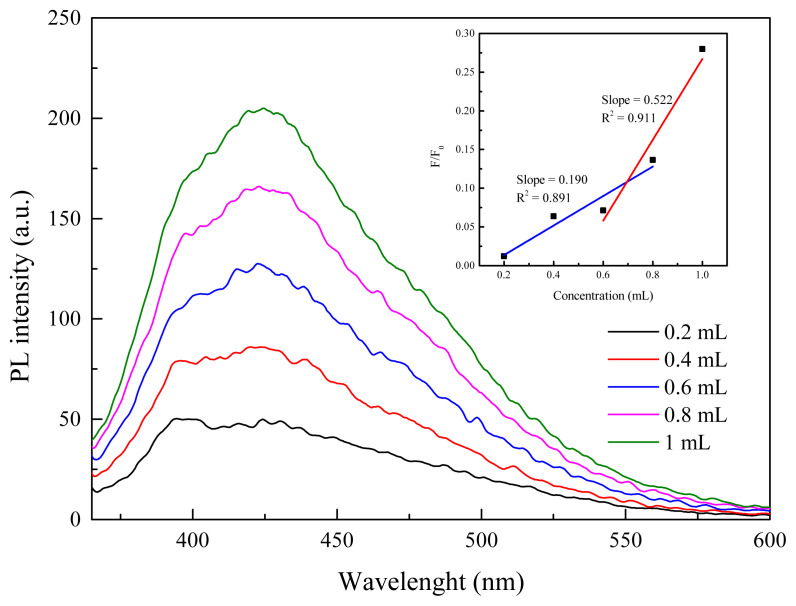
The fluorescence responses of the B-CQD with different concentrations (inset: the relationship between F/F_0_ and the concentrations).

To measure quantum yield (QY) of B-CQDs, coumarin 2 solution was dissolved at 10^−7^ M in ethanol as a reference (*QY_0_=0.68*) [47] and the QY of B-CQDs in deionized water at different concentration. Horiba Scientific’s guide to measurement of fluorescence QY is used for QY calculation [48]. Afterwards, QY of B-CQDs is calculated by following equation (1),


(1) $$QY_{B-CQDs}=QY_{0}\;\left(\frac{m}{m_{0}}\right)\;\left(\frac{\eta^{2}}{\eta_{0}^{2}}\right)$$

where *QY_B-CQDs_* and *QY_0_* are QY of B-CQDs and Coumarin, respectively. *m* is the slope value, and *η* is the refractive index of solvent. Here, *η* and *η_0_* values are 1.333 and 1.36, respectively. Figure 5 depicts the fluorescence responses of the B-CQD with different concentrations. Furthermore, the relationship between F/F_0_ and the concentration is shown in the Figure 5 inset. The QY of B-CQDs is calculated to be 12.41% by using Coumarin 2 as a reference. This result is integrated with previous studies in literature [38,39]. The quantum yields show that the B-CQDs as an electron acceptor can act as a driving force for charge transfer, especially in the excited state [39]. Therefore, it could lead to improve the performance parameters of OSCs.

### 3.2. Optical and morphological characterization of organic photoactive layer

**Figure 6:**
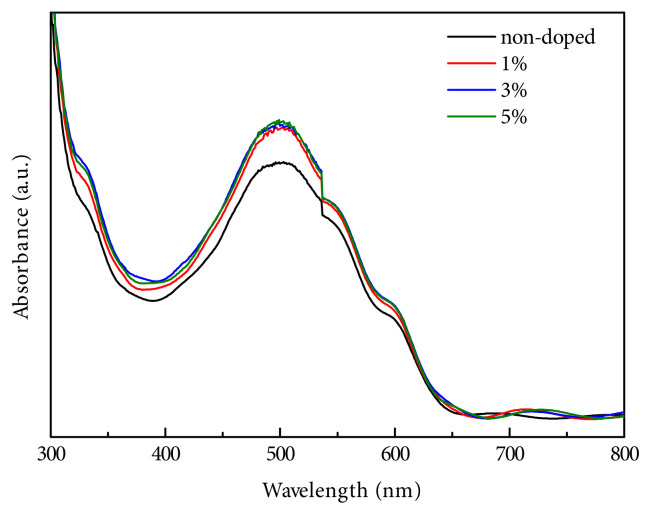
The measured UV-Vis absorption spectra of the non-doped and doped films of P3HT:PCBM.

To understand the addition of B-CQDs into P3HT:PCBM blend, optical absorption spectra and optical band gaps (E_g_) of non-doped and B-CQDs doped films were studied by UV-Vis spectrometer in the range of 300–800 nm. As seen in Figure 6, the optical absorption of P3HT:PCBM films showed similar characteristics and was observed around at 500 nm, which is correlated with previous studies [49,50]. Additionally, the first absorption shoulder was exhibited around at 330 nm referred to PCBM and a dominant peak at 500 nm attributed to P3HT. The dominant peak could be related to one exciton and two photons’ generations [51]. The results also showed that the intensity of absorption peaks increased after addition of B-CQDs into blend.

**Figure 7:**
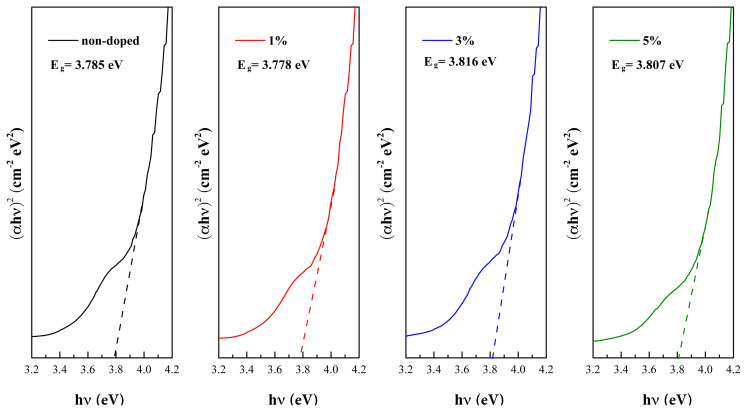
The measured optical band gap extracted from Tauc’s plot of the non-doped and doped films of P3HT:PCBM.

The optical band gaps’ (E_g_) values of P3HT:PCBM films were determined from the intercept of (αhν)^2^ vs. (hν) curves [52], as shown in Figure 7, by the following equation (2):


(2) $${\left(\alpha h\nu \right)}^{2}=A\;(h\nu -Eg)$$

where *α*, hν, and A are the absorption coefficient, photon energy, independent constant, respectively. The average thickness of P3HT:PCBM films was about 200 nm. The E_g_ values were increased from 3.785 eV for non-doped P3HT:PCBM film to 3.816 eV and 3.807 eV for 3 vol.% of B-CQDs and 5 vol.% of B-CQDs, respectively, while it decreased to 3.778 eV for 1 vol.% of B-CQDs, as seen in Table 1.

As known, the E_g_ values of P3HT:PCBM film depend on the concentration of P3HT:PCBM film as well as thickness of P3HT:PCBM film [53,54]. Besides, the electrical parameters of OSC are affected by increased E_g_ values due to the chemical composition of P3HT, good crystallinity, and as a result of the gap states formation. Therefore, it can be stated that there is a correlation between the optical properties and the electrical parameters of OSC [55].

**Figure 8:**
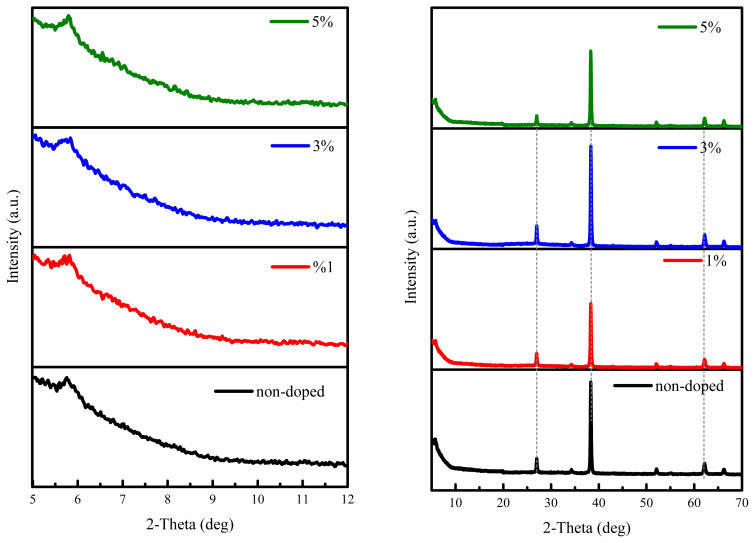
The X-ray diffraction (XRD) spectra of the non-doped and doped films of P3HT:PCBM.

The crystallinity of active layer is important for improving device performance since it does not hinder the charge transport [56]. Therefore, to investigate the crystallinity of non-doped and B-CQDs doped P3HT:PCBM films, the XRD pattern was recorded in the range of 5°–70°. The XRD spectra are presented in Figure 8. As seen in Figure 8(a), the P3HT (100) diffraction peak was observed around a 2 Θ value of 5.4°, while no significant difference corresponding d-space of 1.161 nm between the XRD patterns of non-doped and B-CQDs doped P3HT:PCBM films. It appears that the intensity of the XRD peaks clearly increased after adding 3 vol.% and 5vol.% B-CQDs, while the intensity of XRD peak slightly decreased after adding 1 vol.% B-CQDs, indicating an improvement in crystallite size for addition of 3 vol.% of B-CQDs and 5 vol.% of B-CQDs, as shown in Figure 8(b). The mean sizes of these films’ crystallite were extracted from Scherrer equation, which are also summarized in Table 1 [57]. The results showed that the crystallite sizes of P3HT:PCBM films increased from 38.61 nm for non-doped to 39.99 nm for 3 vol.% and 39.79 nm for 5 vol.% of B-CQDs, respectively, whereas decreased to 37.68 nm for 1 vol.% of B-CQDs. Based on these results, it can be said that CQDs are good candidates for obtaining good crystallites [58]. These results provide that the charge transport properties of P3HT:PCBM films could be improved after the addition of B-CQDs, thus the performance parameters of OSCs tend to improve.

**Figure 9:**
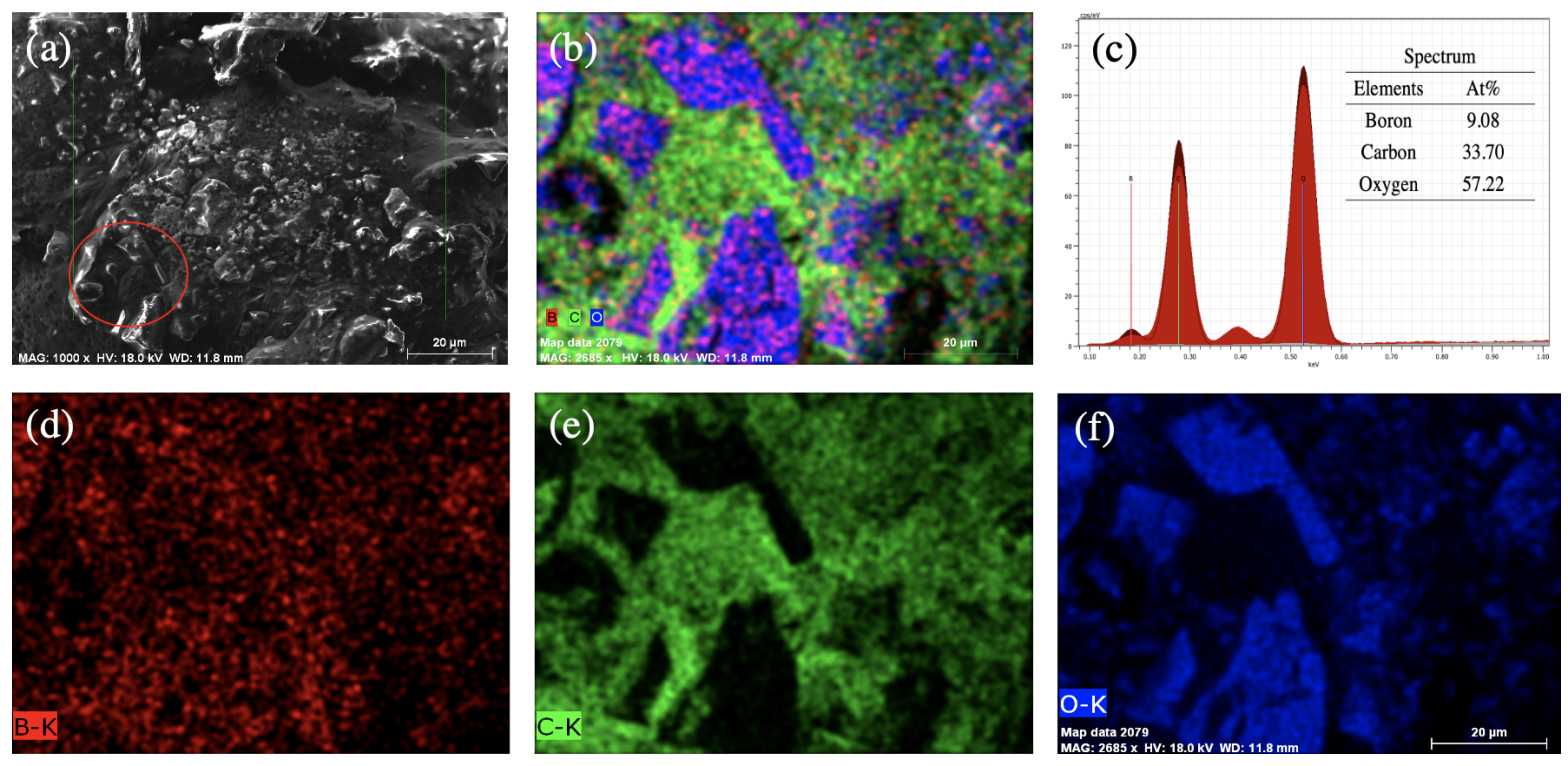
SEM-EDX mapping analysis of B-CQDs. (a) EDX mapping based SEM image of B-CQDs. (b) The elemental mapping of B-CQDs showing, (c) EDX representation of the B-CQDs, the inset image shows the elemental composition percentage in B-CQDs. (d) B-K series, (e) C-K series, and (f) O-K series.

In Figure 9, the presence of boron element in CQDs was performed by SEM-EDX mapping analysis. As shown in Figure 9(b), besides the XRD analysis, EDS mapping were performed for further investigation of the B-CQDs composition. The SEM-EDX results showed that they consist of three elements: oxygen, carbon, and boron at the amount of 57.22%, 33.70% and 9.08%, respectively, as given in Figure 9(c). Oxygen can be associated with the functional groups on the CQDs, while the main element of CQD is carbon. As a result, the presence of boron in the CQDs was clearly observed by EDX analysis.

**Figure 10:**
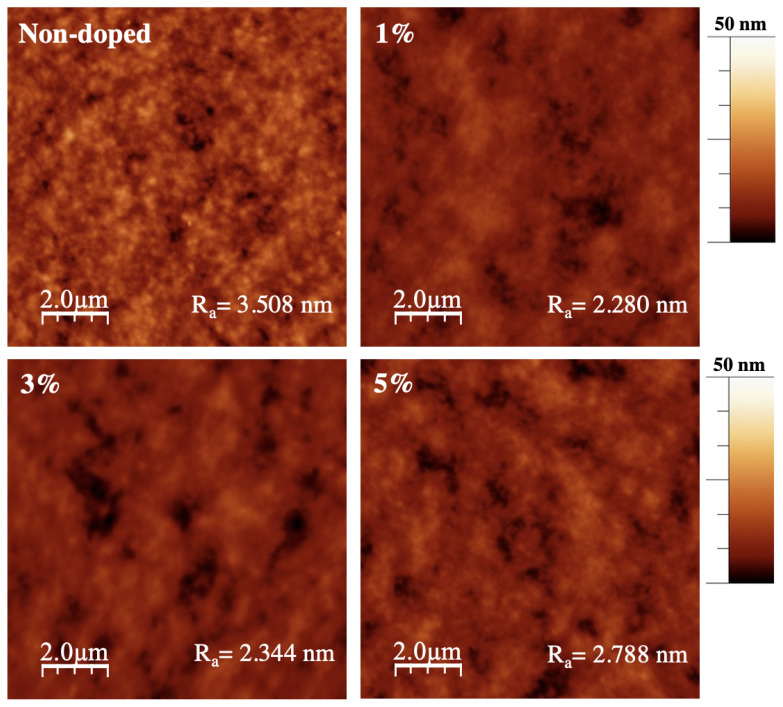
AFM images of the non-doped and doped films of P3HT:PCBM.

The microstructure of organic photoactive layer plays a significant role in the electrical parameters of OSC, thereby, the surface morphology of non-doped and B-CQDs doped P3HT:PCBM films was studied by atomic force microscope (AFM) technique, and all AFM image processing were performed by WSxM 5.0 software [59]. AFM images are presented in Figure 10, and the average roughness (R_a_) values are also summarized in Table 1. As seen, the R_a_ of non-doped and B-CQDs doped P3HT:PCBM film were measured to be 3.508 nm for non-doped film, 2.280 nm for doping 1 vol.% of B-CQDs, 2.344 nm for doping 3 vol.% of B-CQDs, and 2.788 nm for doping 5 vol.% of B-CQDs. The results indicate that a lower surface roughness was obtained after adding B-CQDs. To improve electrical parameter of OSC, the optimization of B-CQDs concentration has a great influence on the charge transport properties of organic photoactive layer due to its surface morphology.

### 3.4. Device characterization

**Figure 11:**
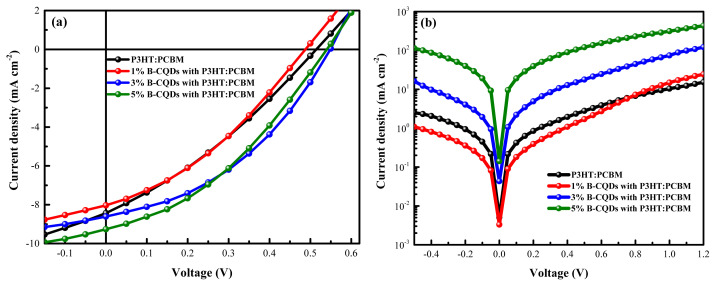
Current density-voltage *(J*–*V)* curves of all the OSCs with various volume of B-CQDs **(a)** under illumination of AM 1.5 G, 80 mW cm^−2^, **(b)** in the dark.

The structure of final device had a configuration with FTO/TiO_2_ (90 nm)/P3HT:PCBM (200 nm)/MoO_3_ (10 nm):Ag (80 nm). To figure out the effect of B-CQDs content on the electrical parameters of OSCs, the devices were fabricated with non-doped and doped of BCQDs into the photoactive layer. The photovoltaic performance of as-prepared devices was measured under light and dark conditions, as shown in Figures 11(a)&(b), and the photovoltaic parameters are also summarized in Table 2.

The non-doped OSC presents a fill factor (FF) of 31.77%, a short circuit current density (J_sc_) of 8.437 mA cm^−2^, open-circuit voltage (V_oc_) of 515 mV, and a lower PCE value of 1.72%. Compared to non-doped OSC, the best photovoltaic performance of OSC doping 3 vol.% B-CQDs was depicted a PCE of 2.33%, which was calculated from FF of 39.65%, V_oc_ of 546 mV, and J_sc_ of 8.606 mA cm^−2^. The increase in short circuit current density (J_sc_) and fill factor (FF) are the main reasons for the improvement of PCE [60]. As known, addition of CQDs is also affected on these parameters in terms of carrier mobility, trap density, carrier lifetime, and diffusion length [61]. Therefore, the effect of doping B-CQDs with various concentration on FF increased from 31.77% to 33.30%, 39.65%, and 36.04% for 1 vol.%, 3 vol.%, and 5 vol.% of B-CQDs doped, respectively. The values of J_sc_ increased from 8.337 mA cm^−2^ to 8.606 mA cm^−2^ and 9.264 mA cm^−2^ for doping 3 vol.% and 5 vol.% of B-CQDs P3HT:PCBM, respectively, while decreased to 8.034 mA cm^−2^ for doping 1 vol.% of B-CQDs. Although there is no big difference between non-doped and 1 vol.% of B-CQDs in terms of FF and J_sc_, a low PCE of 1.62% was observed for 1 vol.% of B-CQDs doped, which is related to a low V_oc_ of 484 mV. Furthermore, the photovoltaic performance of 1 vol.% of B-CQDs doped OSCs may obtaine a lowest PCE because of the factors such as crystallinity of active layer, morphology, and the trap density that can effect V_oc_ [62,63]. These results are also related to the morphological and structural properties of non-doped and B-CQDs doped P3HT:PCBM films.

As is known, the R_s_ plays an important role for determining the FF and the J_sc_. Increasing FF could be associated with obtaining better device performance after the additive of B-CQDs to improve photovoltaic performance. This could be attributed to reduce serial resistance (R_s_) while increase shunt resistance (R_sh_) values [64]. The values of R_s_ and R_sh_ were measured from J-V curves obtained under illuminated condition, which are summarized in Table 2. The R_s_ values with B-CQDs additive showed a dramatical decrease from 30.79 Ω cm^−2^ (non-doped) to 20.53 Ω cm^−2^, to 12.65 Ω cm^−2^ and to 14.46 Ω cm^−2^ with 1 vol.%, 3 vol.%, and 5 vol.% of B-CQDs additive, respectively. Moreover, the R_sh_ values with B-CQDs additive were increased from 97.65 Ω cm^−2^ to 147.62 Ω cm^−2^, to 210.88 Ω cm^−2^ and to 176.55 Ω cm^−2^ with 1 vol.%, 3 vol.%, and 5 vol.% of B-CQDs additive, respectively. The R_sh_ is attributed to leakage current and charge transport properties. Therefore, the lowest 12.65 Ω cm^−2^ of R_s_ and the highest 210.88 Ω cm^−2^ of R_sh_ were obtained from OSCs with 3 vol.% of B-CQDs additive into P3HT:PCBM blend. The results show that the charge transport properties are improved by integrating perfectly with increasing FF values [43].

## 4. Conclusion

In this study, boron doped CQDs were synthesized for use as additive in P3HT:PCBM blend as an photoactive layer to improve photovoltaic parameters. The device with the 3 vol.% B-CQDs additive P3HT:PCBM device showed 39.65% of FF, 546 mV of V_oc_, 8.606 mA cm^−2^ of J_sc_, which led to a 2.33% of PCE (a 35% increasing). In addition, the lowest 12.65 Ω cm^−2^ of R_s_ and the highest 210.88 Ω cm^−2^ of R_sh_ are calculated with 3 vol.% of B-CQDs addition. After the addition of B-CQDs in the photoactive solution, charge transport properties were enhanced. The FF and the J_sc_ values were increased by virtue of an improvement P3HT crystallinity as well as morphological and structural properties. The obtained results indicate that the heteroatom doped CQDs is an excellent candidate to the improvement of highly efficient OSCs.

## Figures and Tables

**Table 1:** The optical band gap, crystal size, and roughness values of non-doped and doped B-CQDs P3HT:PCBM films.

	E_g_ (eV)	Crystallite size (nm)	Roughness (nm)
Non-doped	3.785	38.61	3.508
1 vol.% of B-QCDs	3.778	37.68	2.280
3 vol.% of B-QCDs	3.816	39.99	2.344
5 vol.% of B-QCDs	3.807	39.79	2.788

**Table 2:** Device figure-of-merit parameters of the organic solar cells. Non-doped and doped B-CQDs under illumination of AM 1.5, 80 mW/cm^2^.

	FF (%)	V_oc_ (mA)	J_sc_ (mA cm^−2^)	PCE (%)	R_s_ (Ω cm^−2^)	R_sh_ (Ω cm^−2^)
Non-doped	31.77	0.515	8.437	1.72	30.79	97.65
1 vol.% of B-QCDs	33.30	0.484	8.034	1.62	20.53	147.62
3 vol.% of B-QCDs	39.65	0.546	8.606	2.33	12.65	210.88
5 vol.% of B-QCDs	36.04	0.538	9.264	2.25	14.46	176.55
